# Application of machine learning for risky sexual behavior interventions among factory workers in China

**DOI:** 10.3389/fpubh.2023.1092018

**Published:** 2023-08-03

**Authors:** Fang Zhang, Shiben Zhu, Siyu Chen, Ziyu Hao, Yuan Fang, Huachun Zou, Yong Cai, Bolin Cao, Kechun Zhang, He Cao, Yaqi Chen, Tian Hu, Zixin Wang

**Affiliations:** ^1^Department of Science and Education, Shenzhen Baoan Women's and Children's Hospital, Shenzhen, Guangdong, China; ^2^Centre for Health Behaviours Research, Jockey Club School of Public Health and Primary Care, The Chinese University of Hong Kong, Hong Kong, China; ^3^Department of Health and Physical Education, The Education University of Hong Kong, Hong Kong, China; ^4^School of Public Health, Sun Yat-sen University, Shenzhen, China; ^5^Kirby Institute, University of New South Wales, Sydney, NSW, Australia; ^6^School of Public Health, Shanghai Jiaotong University School of Medicine, Shanghai, China; ^7^School of Media and Communication, Shenzhen University, Shenzhen, China; ^8^Longhua District Center for Disease Control and Prevention, Shenzhen, China

**Keywords:** machine learning, risky sexual behaviors, random forest model, logistic regression, HIV/STIs

## Abstract

**Introduction:**

Assessing the likelihood of engaging in high-risk sexual behavior can assist in delivering tailored educational interventions. The objective of this study was to identify the most effective algorithm and assess high-risk sexual behaviors within the last six months through the utilization of machine-learning models.

**Methods:**

The survey conducted in the Longhua District CDC, Shenzhen, involved 2023 participants who were employees of 16 different factories. The data was collected through questionnaires administered between October 2019 and November 2019. We evaluated the model's overall predictive classification performance using the area under the curve (AUC) of the receiver operating characteristic (ROC) curve. All analyses were performed using the open-source Python version 3.9.12.

**Results:**

About a quarter of the factory workers had engaged in risky sexual behavior in the past 6 months. Most of them were Han Chinese (84.53%), hukou in foreign provinces (85.12%), or rural areas (83.19%), with junior high school education (55.37%), personal monthly income between RMB3,000 (US$417.54) and RMB4,999 (US$695.76; 64.71%), and were workers (80.67%). The random forest model (RF) outperformed all other models in assessing risky sexual behavior in the past 6 months and provided acceptable performance (accuracy 78%; sensitivity 11%; specificity 98%; PPV 63%; ROC 84%).

**Discussion:**

Machine learning has aided in evaluating risky sexual behavior within the last six months. Our assessment models can be integrated into government or public health departments to guide sexual health promotion and follow-up services.

## 1. Introduction

Sexually transmitted infections (STIs) remain a global public health issue, as international absolute incident cases for STIs increased, from 486,770,168 in 1990 to 769,850,699 in 2019 (1). Considering the rising incidence of STIs, WHO has proposed a Global Health Sector Strategy for STIs 2016–2021, which aims to end the public health problem of the STI epidemic by 2030, including reducing the incidence of gonorrhea by 90% globally (2018 global baseline) and in 80% of countries reducing congenital syphilis per 100,000 live births reduce incidence by more than 50 cases (2). Additionally, previous studies suggest that various interventions should increase HIV/STIs awareness and reduce HIV/STIs-related risk behaviors among factory workers in China because they are particularly vulnerable to infection (3–5). One key strategy to reduce the incidence of HIV/STIs is to increase awareness and attitudes toward risky sexual behaviors (6, 7). Barriers to raising awareness and attitudes toward risky sexual behavior include uneven regional development, limited knowledge availability, and economic costs (8, 9). Screenings can effectively increase health screenings' acceptance, usability, and acceptability among users (10, 11). Estimating the probability of risky sexual behaviors in individuals or groups can help with targeted education and reduce financial costs.

Machine learning is uniquely suited to developing predictive models, automatically learning from complex, non-linear big data without statistical inference or assumptions, and achieving high accuracy (12, 13). Machine learning models have been widely used to predict the future risk of diseases such as Alzheimer's disease (14), COVID-19 (15), and Leukemia (16). A study in 2021 shows that machine learning can be used to build effective prediction model(s) to identify adolescents likely to engage in HIV/STIs risk behaviors (17). Another study from Zimbabwe reports that machine learning models can help identify individuals at high risk of HIV infection with 85% accuracy, assist policymakers in developing targeted HIV/STIs prevention and screening strategies, and inform sociodemographic and risk behavior data (18). However, building a follow-up database is time-consuming and expensive, factory workers are highly mobile, and the questions are too private to guarantee quality. We always need to quickly assess the probability of individual or group risky sexual behaviors occurring at the point so that we can take targeted educational measures. To our knowledge, there are currently no estimation models based on cross-sectional studies to evaluate risky sexual behaviors of individuals or groups.

This study aimed to seek the best-performing algorithm and evaluate risky sexual behaviors in the past 6 months using machine learning models. At the same time, we understood that the impact of the top 30 risk factors that achieved similar ROC values on risky behaviors would help inform decisions about prevention, treatment, service delivery, and resource allocation.

## 2. Materials and methods

### 2.1. Study data for risky sexual behaviors estimation in the past 6 months

This study was a cross-sectional study that collected data from full-time factory workers. Employees over 18 years old and being full-time employed from the randomly selected workshops were invited to visit Longhua District Center for Disease Control and Prevention (CDC). At the CDC, our trained fieldworkers briefed the study to the eligible 2,700 workers from the selected workshops. Of these workers, 2023 completed a self-administered questionnaire. Participants were guaranteed anonymity and the right to quit at any time without consequence. Written informed consent was obtained from all participants. Participants who completed our survey received a cash coupon of ¥20 (US$2.60). The dataset used in this study was built between October and November 2019 and collected from 2023 adult factory workers in 16 different factories. We used data from Longhua District Center for Disease Control and Prevention (CDC) to develop and validate the machine learning model. Sixteen factories were randomly selected, including four machining factories, three electronic equipment factories, three printing and dyeing factories, two chemical raw material factories, one smelting factory, one garment factory, one food and beverage factory, and another factory. Questionnaires collected were composed of sociodemographic information, lifestyle habits, clinical health, psychological status, and risky sexual behaviors. The questionnaire was designed by a team of two public health researchers, epidemiologists, health psychologists, health communication specialists, and a factory worker. Twenty workers were invited to participate in the pilot study under the guidance of trained researchers. These 20 workers did not participate in the follow-up survey. The questionnaire was revised and finalized based on their feedback. The study was carried out following the guidelines of the Declaration of Helsinki and was approved by the ethics committees of the School of Public Health, Sun Yat-sen University (2019/3).

### 2.2. Evaluation factors for risky sexual behaviors prediction

Risky sexual behaviors were defined as having sexual intercourse without a condom in the last 6 months. We extracted all items from the dataset as variables, yielding 250 categorical and 15 continuous variables. All variables had no missing values. Categorical variables were encoded to integers using the LabelEncoder from scikit-learn, and then, all variables were normalized by scaling each item to a given range. After different models' training and testing, we found that the model's ROC values changed <1% when the fit was repeated after selecting at least the top 30 features of the optimal model through loops and judgment statements. Finally, the top 30 features of the best performance model were selected as the last evaluation factors. All models would be trained and tested again with 30 new evaluation factors. Although a correlation heatmap by seaborn showed that the correlations between the variables were high, they would not affect our evaluation model and the discovery of certain important factors that could be controlled artificially, so we did not perform a correlation analysis.

### 2.3. Model development and validation for risky sexual behaviors

We randomly divided the data into an 80% training set for model development and a 20% test set for model testing. The study considered six machine learning models, including (1) logistic regression (LR), (2) support vector machines (SVM), (3) random forests (RF), (4) gradient descent boosting models (XGBoost), (5) K-Nearest Neighbor (KNN), (6) Naïve Bayes (NB), (7) neural networks (NN), from Python package “Scikit-sklearn.” LR is an example of supervised learning used to calculate or predict the probability of a binary event occurring. SVM algorithm aims to find a hyperplane in N-dimensional space (number of N features) that classifies the data points explicitly. RF are another popular machine learning algorithm for regression and classification problems. The XGBoost uses a series of decision trees to make predictions and represent an interpretable model. Unlike logistic regression, this model can include higher-order interactions and considers the complex non-linear relationship between the model variables and the outcome. The gradient descent boosting method is extreme gradient boosting, known as XGBoost. XGBoost includes a measure of the model's accuracy with specific variables added, and higher gain values mean it is more critical in generating predictions. KNN algorithm is a simple, easy-to-implement supervised machine learning algorithm that can solve classification and regression problems. NB algorithm is one of the fast and easy ML algorithms to predict a class of datasets. NN algorithm is a machine learning technique that connects layers of nodes (neurons) like a human brain to simulate output.

### 2.4. Measuring models performance

We evaluated the model's overall predictive classification performance using the area under the curve (AUC) of the receiver operating characteristic (ROC) curve. The performance of the model was evaluated in terms of accuracy (the proportion of all observations in the unseen test set that were correctly classified by the algorithm), sensitivity (the proportion of known positive results in the unseen test set that were correctly identified as positive by the algorithm), positive predictive value (PPV; also known as precision; the proportion of positive results predicted by the algorithm that correspond to known positive results in the unseen test set) and specificity (the proportion of known negative results in the unseen test set that were correctly identified as unfavorable by the algorithm). Seen in the test set of known negative results was evaluated. A range of 0.5 or less indicated no predictive power, while 1.0 indicated perfect predictive power.

### 2.5. Statistical analysis

We applied a univariate approach to describe the background variables. At first, seven models were trained to obtain a best model by ROC value. The loop was then repeated to obtain the optimal number of variables so that the ROC value of the best model decreased by <1%. The top thirty variables with the highest importance in the best model were taken and trained again with seven models to obtain their statistics separately. All analyses were performed using the open-source Python version 3.9.12.

## 3. Results

### 3.1. Background characteristics of the participants

Our study included 2023 factory workers, of whom 1,027 male workers had no risky sexual behavior, 334 male workers had risky sexual behavior, 498 female workers had no risky sexual behavior, and 164 female workers had risky sexual behavior in the past 6 months. The average age of factory workers without risky sexual behavior was 30.73 years, and the average age of factory workers with risky sexual behavior was 32.63 years. The majority of them were the Han majority (84.53%), without permanent residency in other provinces (85.12%) or Rural (83.19%), junior high school (55.37%), with monthly personal income from ¥3,000 (US$417.54) to ¥4,999 (US$695.76) (64.71%), and workers (80.67%). About half of the participants had no children (44.64%), were married (53.75%), and in electronic equipment (41.35%). Further details are given in Table 1.

**Table 1:** Characteristics of background variables stratified by risky sexual behaviors in the past 6 months (*N* = 2023).

	**No risky sexual behaviors (*n* = 1,525)**	**Risky sexual behaviors (*n* = 498)**
Age ($\bar{x}+sd$)	30.73 ± 8.44	32.63 ± 8.32
**Gender**		
Male	1,027 (50.77%)	334 (16.51%)
Female	498 (24.62%)	164 (8.11%)
**Ethnic group**		
Han	1,280 (63.27%)	430 (21.26%)
Others	245 (12.11%)	68 (3.36%)
**Hometown**		
Shenzhen	16 (0.79%)	13 (0.64%)
Other cities in Guangdong	198 (9.79%)	74 (3.66%)
Other provinces	1,311 (64.80%)	411 (20.32%)
**Hometown attributes**		
Big cities	35 (1.73%)	8 (0.40%)
Small and medium-sized cities	212 (10.48%)	85 (4.20%)
Rural	1,278 (63.17%)	405 (20.02%)
**Marital status**		
Single	531 (26.25%)	89 (4.4%)
Have a boyfriend/girlfriend	141 (6.97%)	42 (2.08%)
Married	748 (36.97%)	339 (16.76%)
Divorce/widowhood	40 (1.98%)	12 (0.59%)
Others	65 (3.21%)	16 (0.79%)
**Number of children**		
0	744 (36.78%)	159 (7.86%)
1	305 (15.08%)	152 (7.51%)
2	378 (18.69%)	155 (7.66%)
3	83 (4.1%)	29 (1.43%)
4	12 (0.59%)	3 (0.15%)
5	3 (0.15%)	0 (0.0%)
**Educational background**		
Primary school and below	43 (2.13%)	28 (1.38%)
Junior high school	849 (41.97%)	271 (13.4%)
High school	535 (26.45%)	173 (8.55%)
Junior college	80 (3.95%)	23 (1.14%)
Bachelor's degree or above	18 (0.89%)	3 (0.15%)
**Salary (RMB/per month)**		
< 1,000	26(1.29%)	5 (0.25%)
1,000–2,999	108 (5.34%)	44 (2.17%)
3,000–4,999	1,005 (49.68%)	304 (15.03%)
5,000–6,999	352 (17.4%)	133 (6.57%)
7,000–9,999	28 (1.38%)	10 (0.49%)
>10,000	6 (0.3%)	2 (0.1%)
**Factory type**		
Smelting	30 (1.48%)	18 (0.89%)
Chemical raw materials	120 (5.93%)	29 (1.43%)
Printing and dyeing	58 (2.87%)	27 (1.33%)
Printing and dyeing	18 (0.89%)	7 (0.35%)
Clothing textile	53 (2.62%)	23 (1.14%)
Machining	194 (9.59%)	81 (4.00%)
Electronic equipment	734 (36.28%)	224 (11.07%)
Food processing	46 (2.27%)	20 (0.99%)
Others	272 (13.45%)	69 (3.41%)
**Professional role**		
Workers	1,234 (61.0%)	398 (19.67%)
Team leader	106 (5.24%)	36 (1.78%)
Section chief	12 (0.59%)	1 (0.05%)
Workshop director	10 (0.49%)	7 (0.35%)
Office clerk	64 (3.16%)	23 (1.14%)
Senior management	6 (0.3%)	2 (0.1%)
Others	93 (4.6%)	31 (1.53%)

### 3.2. Different models' performance and top 30 factors of the random forest model

**Figure 1:**
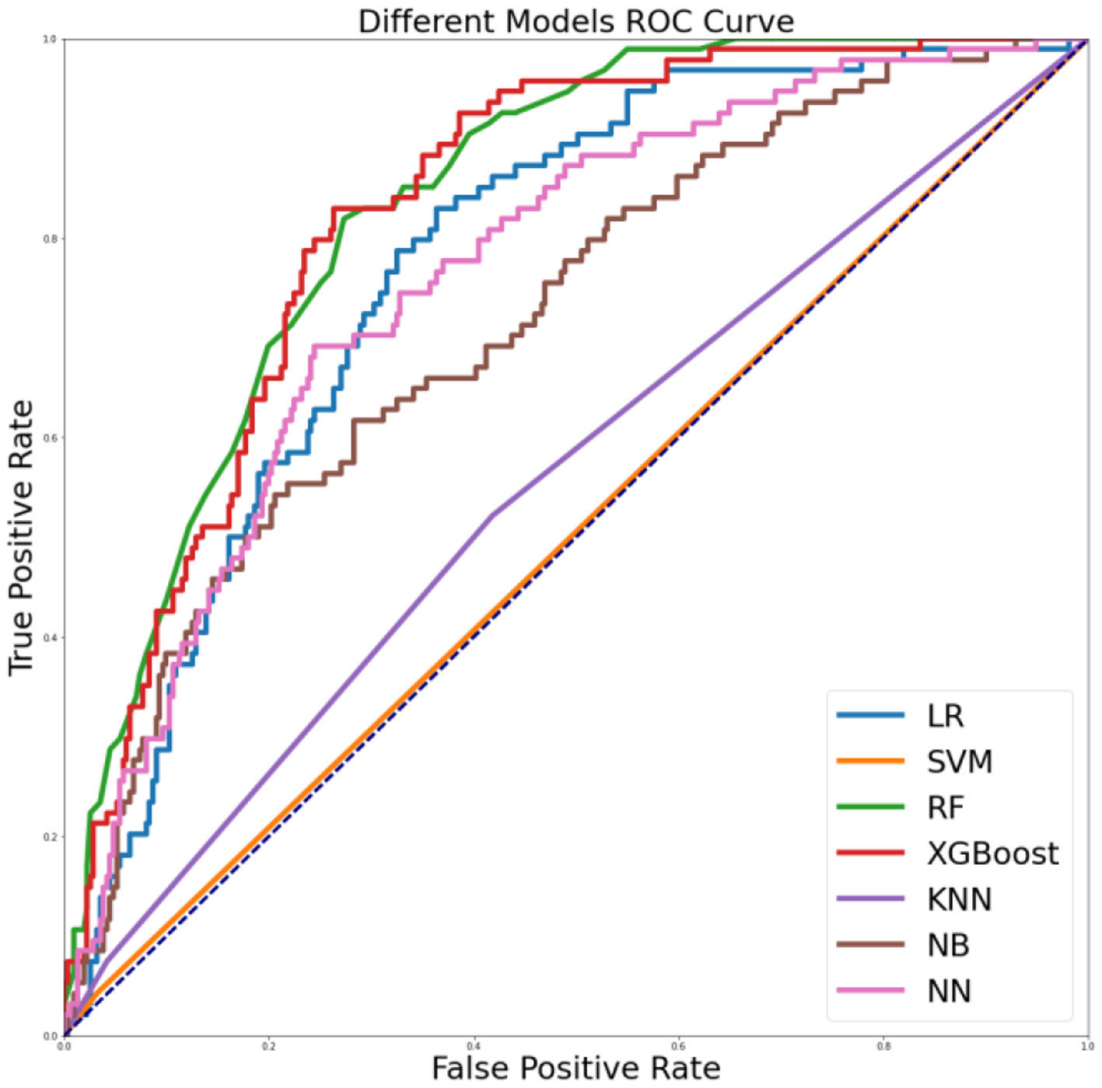
ROC curves for different machine learning models on the risky sexual behaviors in the past 6 months.

We found seven machine learning models including LR, SVM, RF, XGBoost, KNN, NB, and NN with ROC values>51% (range 51–84%). Of the developed machine learning models, the random forest model (RF) outperformed all other models and provided acceptable performance in evaluating risky sexual behaviors in the past 6 months (Accuracy 78%; Sensitivity 11%; Specificity 98%; PPV 63%; ROC 84%), followed by the XGBoost model (Accuracy 79%; Sensitivity 44%; Specificity 89%; PPV 55%; ROC 83%). Details are provided in Figure 1 and Table 2.

**Table 2:** Different machine learning models' performance.

**Name**	**Accuracy**	**Sensitivity**	**Specificity**	**PPV**	**ROC**
LR	0.77	0.35	0.89	0.50	0.77
SVM	0.75	0.04	0.97	0.29	0.51
RF	0.78	0.11	0.98	0.63	0.84
XGBoost	0.79	0.44	0.89	0.55	0.83
KNN	0.75	0.07	0.96	0.35	0.56
NB	0.75	0.47	0.84	0.46	0.72
NN	0.76	0.40	0.87	0.49	0.76

**Figure 2:**
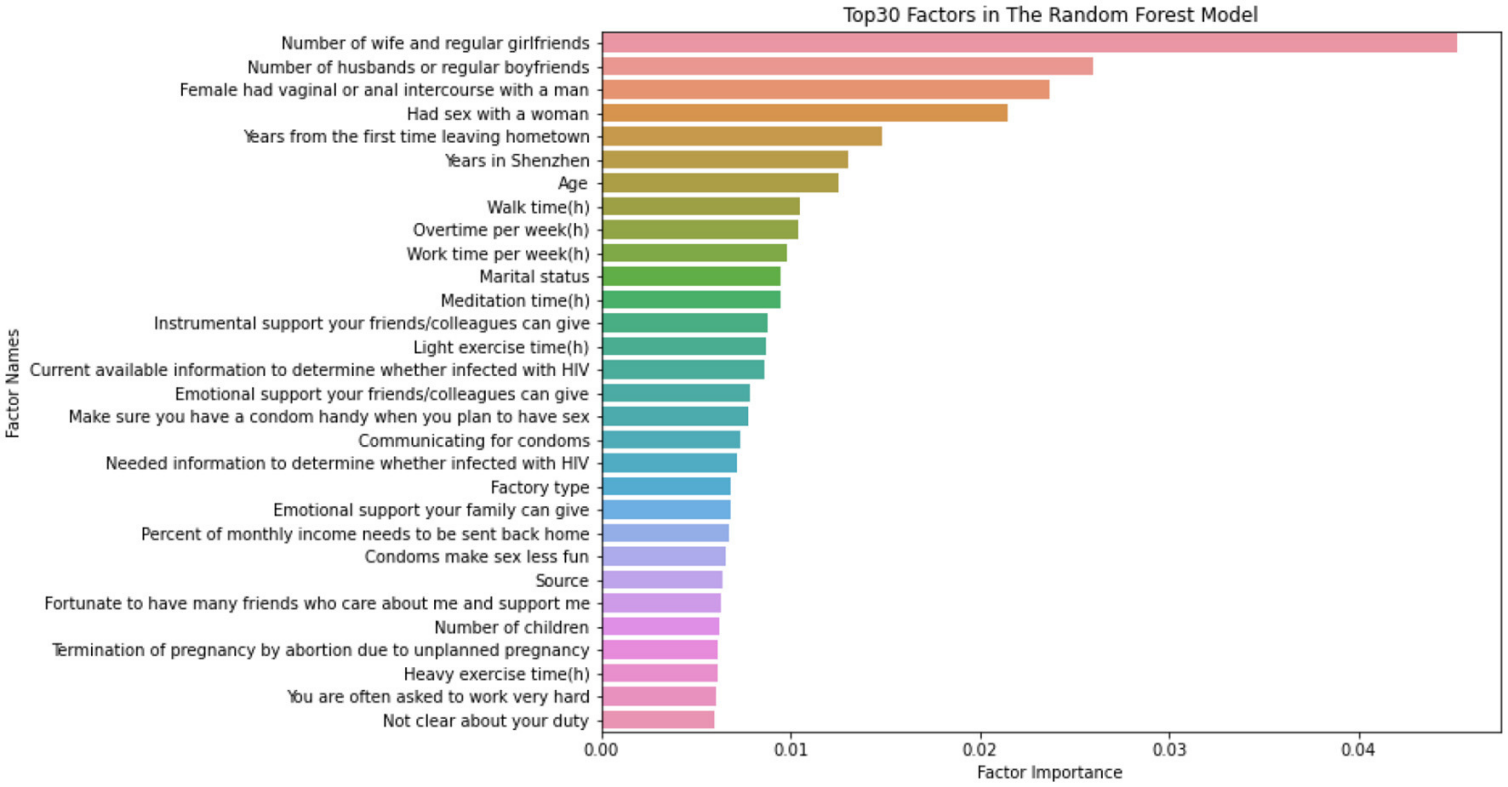
Top 30 factors in the random forest model.

The above section shows that the random forest model achieved the best performance. The top 30 important evaluation factors in the random forest model for evaluating risky sexual behaviors in the past 6 months were identified. The rank of these factors is listed in Figure 2.

### 3.3. Different models' performance with the top 30 factors of the random forest model

**Figure 3:**
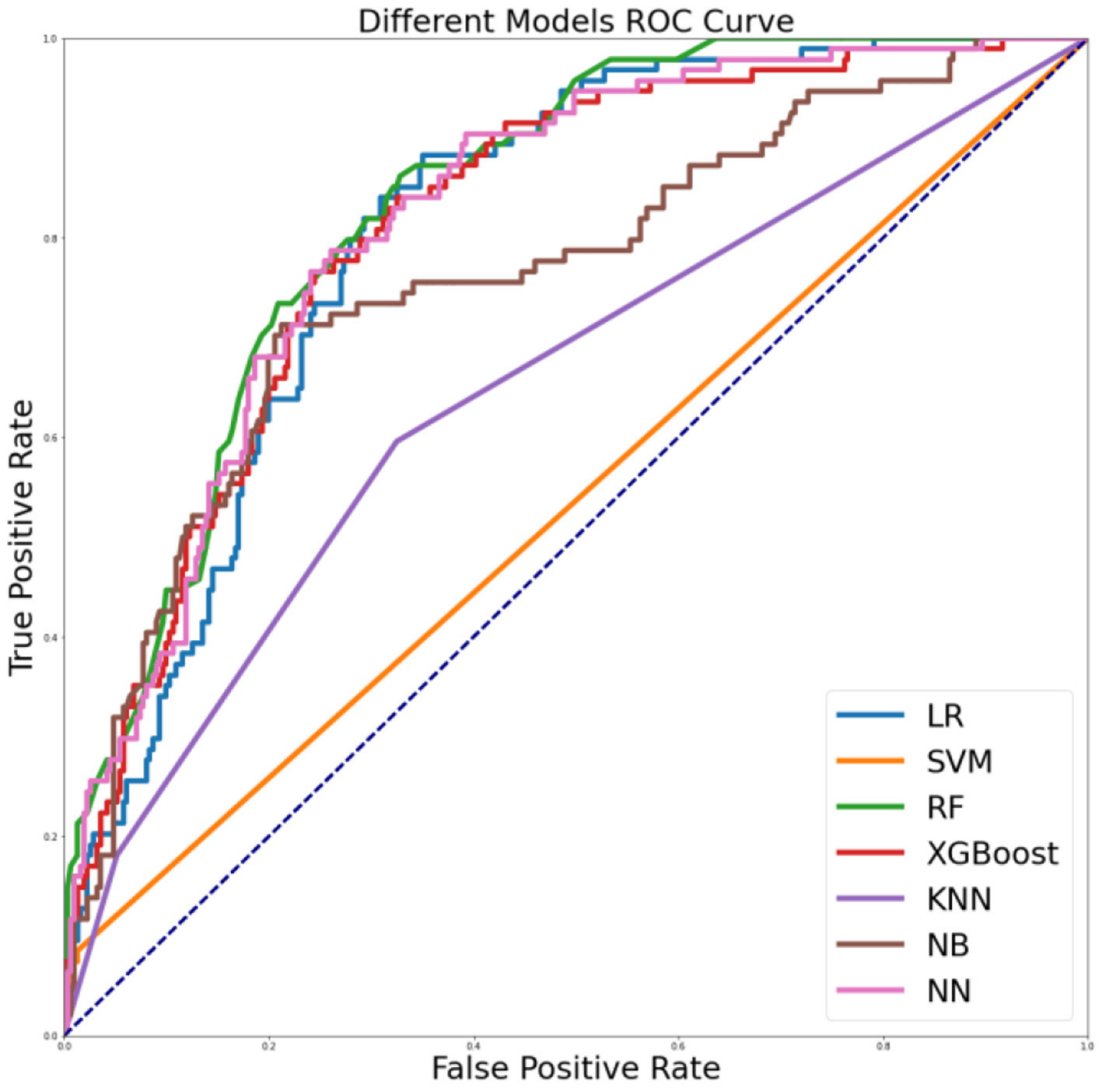
ROC curves for different machine learning models with the top 30 factors of the random forest model.

We trained seven machine learning models with the top 30 factors and achieved ROC values>54% (range 54–84%). Overall, the results from the top 30 factors reveal that the random forest model had a ROC value of 84%, meaning that it could better evaluate risky sexual behaviors, as shown in Figure 3 and Table 3.

**Table 3:** Different machine learning models' performance with the top 30 factors of the random forest model.

**Name**	**Accuracy**	**Sensitivity**	**Specificity**	**PPV**	**ROC**
LR	0.77	0.26	0.92	0.50	0.81
SVM	0.78	0.07	0.99	0.70	0.54
RF	0.79	0.34	0.92	0.57	0.84
XGBoost	0.78	0.44	0.88	0.53	0.81
KNN	0.77	0.18	0.95	0.52	0.65
NB	0.80	0.34	0.94	0.62	0.76
NN	0.77	0.40	0.88	0.51	0.82

## 4. Discussion

About a quarter of factory workers have had risky sexual behavior in the past 6 months. Among these factory workers, characteristics such as Han Chinese, other provinces, rural areas, junior high school education, married, earning ¥3,000–4,999 (US$417.54–695.76) per month, electronics factories, having nearly 50% of their income to send home, and workers dominated. To our knowledge, this is the first study to use machine learning algorithms to assess the risky sexual behaviors of individuals or groups. Random forests show higher predictive accuracy than classical multivariate logistic regression models in assessing the risk of risky behaviors. Our random forest model performed well in assessing risky behaviors but not in assessing the sensitivity of risky behaviors. Our findings suggest that machine learning-based approaches can help assess risky sexual behavior situations based on various types of questionnaire data collection often encountered in real life. Our machine learning model has potential value as a behavioral intervention tool that could be incorporated into government or public health departments to assess people with high-risk behaviors for early intervention.

Our results found that years from the first-time leaving hometown, workload, and exercise time were important estimators of risky sexual behaviors. In addition, our results suggest the need for further research to investigate the difference between the first-time leaving hometown, workload, and exercise time on risky sexual behaviors in different populations. Based on this finding, the government and the department of public health could target education to reduce risky sexual behaviors and HIV/STIs incidence. Our study demonstrates that a machine learning-based approach effectively evaluates risky sexual behaviors. The machine learning models can potentially promote government and factory policy reform, which will help reduce the incidence of HIV/STIs and initiate preemptive interventions in sexual health promotion. Our study discovers that more exercise and meditation time will lead to risky sexual behaviors in the past 6 months. A previous study found that education combined with community intervention reduced the proportion of workers with risky sexual behaviors and enhanced HIV knowledge (3). This study also suggested that policy intervention combined with peer education enhanced HIV knowledge, perceived condom accessibility, and condom use with regular partners (3). Therefore, target education is more necessary.

Emerging machine-learning methods have the potential to help with medical outcomes (19). Our machine learning models suggest that data collected by conventional questionnaires have some estimative value for evaluating risky behavior. Our variable importance ranking results found that the number of regular sexual partners was critical in assessing the risk of risky sexual behavior. In addition, we found that interventions for leaving hometown, physical activity, and workload were more effective for the onset of risky sexual behaviors. Assessing risky behaviors allows for timely interventions to improve education and early intervention for at-risk populations. Our findings suggest that machine learning models using routine questionnaire data from real-life settings can assess risky sexual behaviors. Our machine learning models are trained using routine questionnaire data and can be translated into sexual health service products or health intervention tools for government personnel. Our machine learning model provides a powerful potential tool for evaluating risky sexual behaviors using data collected from standard questionnaires in an actual environment.

This study has some limitations. This study uses machine learning techniques to provide insight into some factors in evaluating risky sexual behaviors. Its findings must be considered in the context of public health policy, strategy, and practice. However, the sampling technique of the cross-sectional design and the resulting distribution of demographic characteristics may have prevented all relevant factors associated with evaluating risky sexual behaviors among factory workers from being identified. Cross-sectional studies do not show the temporal relationship between exposure and outcome in the same way as longitudinal studies. It may also be underpowered in detecting differences in certain variables. Therefore, a more comprehensive range of studies with larger sample sizes that give higher statistical power may be needed to explore all potentially relevant variables fully. All questionnaire data (condom use, history of HIV testing, psychological status) were self-reported and may be subject to social desirability bias. Although interviewers received technical training to reassure participants and support accurate reporting of dates and events, self-reported data are still vulnerable to recall and social desirability bias regarding sensitive topics. Those recruited and agreed to participate may have been a self-selected group more willing to disclose their sexual behaviors. The survey is limited to the Shenzhen factory and does not reflect the activities of the entire Chinese population.

## 5. Conclusion

Our study shows that random forest models can properly assess risky sexual behaviors over the past 6 months. Based on data from the questionnaire, this risk assessment tool could also be incorporated into government and public health departments to facilitate targeted education to reduce risky sexual behaviors.

## Data availability statement

The original contributions presented in the study are included in the article/supplementary material, further inquiries can be directed to the corresponding author.

## Ethics statement

The study was carried out following the guidelines of the Declaration of Helsinki and was approved by the Ethics Committee of the School of Public Health, Sun Yat-sen University (2019/03).

## Author contributions

FZ, SZ, SC, ZH, and ZW: conceptualization. FZ, KZ, HZ, YCa, BC, and ZW: methodology. FZ, KZ, HC, YCh, and TH: data curation. FZ, SC, ZH, and SZ: formal analysis. FZ, SC, ZH, YF, YCa, BC, HC, YCh, TH, and SZ: project administration. KZ, HZ, and ZW: supervision. FZ, SZ, and SC: writing—original draft preparation and writing—review and editing. All authors have read and agreed to the published version of the manuscript.
